# Positive cerebrospinal fluid in the 2024 McDonald criteria for multiple sclerosis

**DOI:** 10.1016/j.ebiom.2025.105905

**Published:** 2025-09-17

**Authors:** Florian Deisenhammer, Harald Hegen, Georgina Arrambide, Brenda L. Banwell, Tim Coetzee, Sharmilee Gnanapavan, Xavier Montalban, Hayrettin Tumani, Maria A. Willrich, Mark S. Freedman

**Affiliations:** a Department of Neurology, Innsbruck Medical University, Innsbruck, Austria; b Multiple Sclerosis Centre of Catalonia and Department of Neurology-Neuroimmunology, Hospital Universitari Vall d'Hebron, Universitat Autònoma de Barcelona, Barcelona, Spain; c Department of Pediatrics, Johns Hopkins University, Baltimore, USA; d National Multiple Sclerosis Society, New York, NY, USA; e Blizard Institute, Barts and the London School of Medicine and Dentistry, Queen Mary University of London, London, UK; f Department of Neurology, University Hospital of Ulm, Ulm, Germany; g Mayo Clinic, Rochester, MN, USA; h Department of Medicine, University of Ottawa, The Ottawa Hospital Research Institute, Ottawa, Ontario, Canada; i Universitat de Vic/Central de Catalunya (UVic-UCC), Spain

**Keywords:** Cerebrospinal fluid, Free light chains, Multiple sclerosis, Diagnosis, Criteria

## Abstract

The 2024 McDonald diagnostic criteria for Multiple Sclerosis (MS) introduce kappa free light chains (κ-FLC) detection in cerebrospinal fluid (CSF) which can be used interchangeably with oligoclonal IgG bands (OCB) to demonstrate intrathecal immunoglobulin synthesis. Diagnostic sensitivity and specificity of κ-FLC is equal to OCB on a 95% confidence level. In rare cases determination of both, κ-FLC and OCB should be considered as the concordance rate is around 90%. We recommend calculating the κ-FLC index with values of ≥6.1 performing best for diagnosing MS. Validated turbidimetric or nephelometric assays should be applied for which proficiency testing programs are available. There is some prognostic use of the κ-FLC index with higher values predicting higher disease activity. Neurofilament light (NfL) should not be used for diagnostic purposes although it might be useful for prognosis and disease monitoring. All recommendations apply to paediatric and adult relapsing as well as progressive onset MS.

## Introduction

### Some general CSF considerations

Despite further developments in the field of structural and functional imaging methods, the analysis of cerebrospinal fluid (CSF) remains an important diagnostic tool. CSF analysis, including liquid biopsy, provides easy access to certain regions of the central nervous system (CNS) with the advantage to directly detect pathophysiological processes.^1^, ^2^, ^3^ The reliable diagnosis of CNS diseases is often difficult in the early stages due to an overlap of clinical and imaging abnormalities among the differential diagnoses. This is true especially in those diseases with a subacute-chronic course similar to multiple sclerosis (MS) such as: neuromyelitis optica spectrum disorders (NMOSD), myelin oligodendrocyte glycoprotein antibody-associated disease (MOGAD), subacute neuro-infections, and autoimmune encephalitis. As different therapeutic approaches are used for these other conditions, early and accurate diagnosis is crucial for further clinical management.

Analysing CSF from patients with suspected MS provides the opportunity to investigate the inflammatory aspects of the disease and fulfil the essential requirement of the diagnostic criteria for MS for ruling out other differential diagnoses.^4^, ^5^, ^6^, ^7^ Some routine CSF findings (e.g., a high count of inflammatory cells, very low glucose, or very high protein) help indicate conditions other than MS. In addition, intrathecally produced IgG or IgM, together with MRI, help to identify patients with a high probability of developing more active disease or progression and could therefore provide both, prognostic and diagnostic information.^8^^,^^9^ Furthermore, the significance of normal CSF findings (red flag) is crucial in differentiating MS from psychiatric, vascular, and other disorders that can be misdiagnosed as MS.^10^ Likewise, normal CSF findings are important in the context of non-specific MRI findings, which can also contribute to the misdiagnosis of MS.^11^

The sensitive detection of intrathecal immunoglobulin synthesis can be used for early diagnostic confirmation of inflammation in the CNS. In particular, increased CSF IgG synthesis relative to that of serum immunoglobulins (increased IgG index and/or intrathecally produced oligoclonal IgG bands - OCBs type 2 or 3 patterns) was considered typical in patients with MS.^4^^,^^5^ The IgG-Index has been previously regarded as a quantitative and easy to standardise alternative to OCB. However, because of its much lower diagnostic sensitivity it did not prove useful in clinical practice and is no longer recommended.^4^ The availability of immunoglobulin free light chains (FLC) now fills this gap by providing a highly sensitive and easy to standardise diagnostic test.

The interest in FLC as a sensitive detection of a humoural immune reaction in the CNS increased with the improved automated analyses.^12^^,^^13^ Measurement of FLC can now be fully automated with absolute concentrations available within 30 min. This contrasts with the more labour-intensive methodology to detect CSF-specific OCB, taking some 4–5 h, providing only qualitative read-outs by visual inspection. Thus, FLC methodologies are much more cost-effective.^14^

**Fig. 1:**
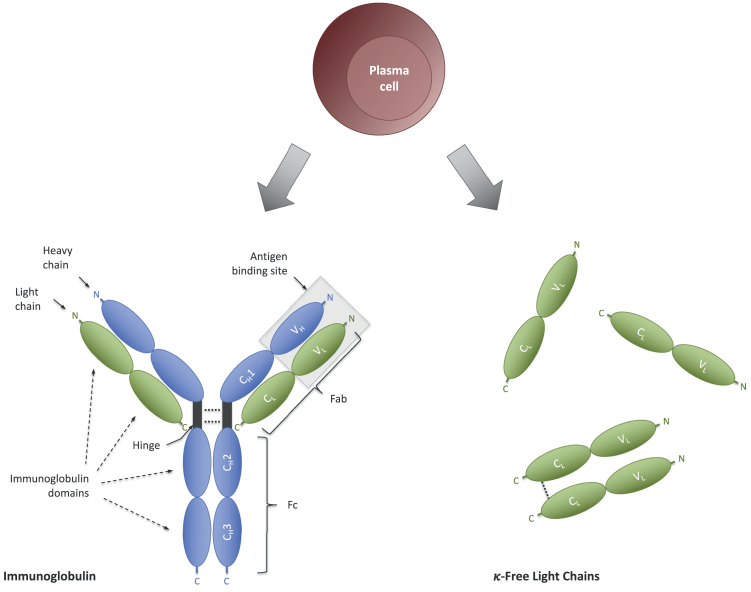
κ-Free light chains and immunoglobulins as marker of intrathecal plasma cell activity. During assembly of immunoglobulins light chains are secreted in excess compared to heavy chains independent of the subtype. Modified from.^15^

Immunoglobulin molecules consist of two heavy and two light chains, each with a constant and a variable part. There are two types of light chains, kappa and lambda, which are synthesised in excess compared to heavy chains by plasma cells and thus occur freely in blood as FLCs (Fig. 1). FLCs in serum have a rather short half-life (2–4 h) due to rapid clearance via the kidneys. In contrast, FLCs produced in the intrathecal space are not subject to active clearance but rather to pressure dependent passive elimination by bulk flow resulting in a half-life comparable to other CSF proteins. Therefore, even a low fraction of intrathecal FLC alongside a small fraction from the blood will result in a relatively large proportion of FLC in the CSF, i.e., a potential marker for intrathecal immunoglobulin synthesis.

It was recognised early on that the detection of intrathecal FLC synthesis is a diagnostically sensitive test for CNS inflammation, which can even be helpful in OCB-negative patients to demonstrate intrathecal inflammation.^16^ Meanwhile it is well accepted, that similar to OCB, intrathecal FLC synthesis shows a high diagnostic sensitivity in patients with MS comprising relapsing and progressive onset as outlined below.^14^^,^^17^ We will focus on the FLC kappa (κ-FLC) subunit, because of its substantially greater diagnostic power and that most of the literature on MS concerns this subunit.^18^^,^^19^

In addition to κ-FLC, we will touch on neurofilament light briefly as it was a topic raised and discussed during the preparation of the 2024 McDonald criteria.

## Oligoclonal bands

**Fig. 2:**
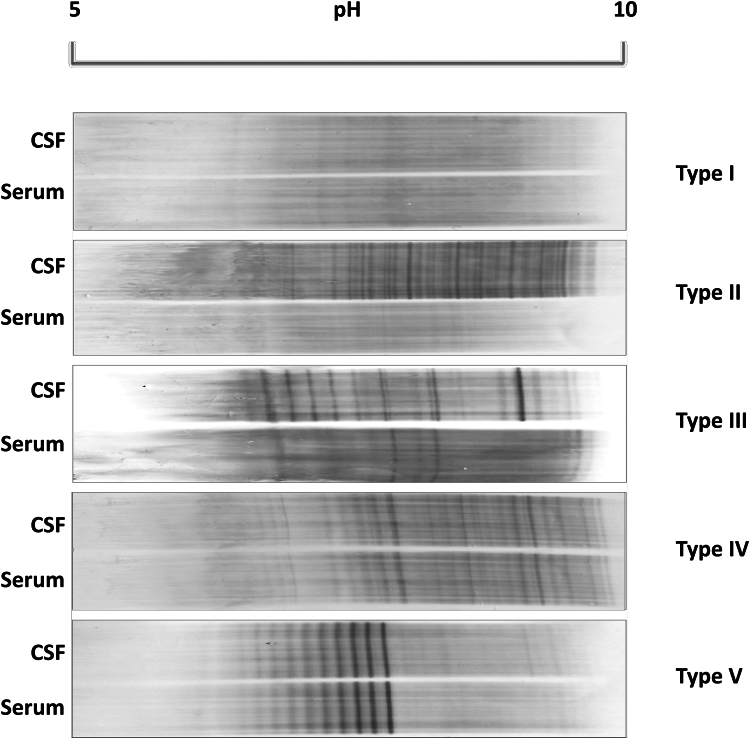
Isoelectric focussing on polyacrylamide gels followed by IgG immunoblotting. OCB typing according to Freedman et al.^4^ Type 1: No bands in CSF and serum. Type 2: Oligoclonal IgG-bands in CSF, not in serum, indicative of intrathecal IgG-synthesis. Type 3: Oligoclonal bands in CSF (like type 2) and additional identical oligoclonal bands in CSF and serum (like type 4), still indicative of intrathecal IgG-synthesis. Type 4: Identical oligoclonal bands in CSF and serum illustrative of a systemic not intrathecal immune reaction, with a leaky or normal BCB and bands passively transferred into the CSF. Type 5: Monoclonal bands in CSF and serum; this is the pattern seen due to the presence of a paraprotein (monoclonal IgG component).

Oligoclonal bands (OCBs) represent discrete bands of immunoglobulins, predominantly clonally expanded IgG (but in some cases, also IgM), detected in the CSF.^20^ OCBs can originate from systemic sources, where identical bands appear in both CSF and serum, indicating systemic clonal expansion, or from intrathecal sources, where unique bands are present only or in greater number in CSF, suggestive of intrathecal IgG synthesis,^21^^,^^22^ as reflected by type 2 and 3 patterns as shown in Fig. 2. These patterns are particularly consistent with MS, detected in over 90% of clinically definite cases.^4^ OCBs are less commonly observed in other conditions such as NMOSD, in 25% or less patients,^23^^,^^24^ and may be a transient phenomenon in this condition. Similarly, OCB can be detected in only around 10% of patients with MOGAD.^25^

In MS, OCBs are a hallmark feature and therefore, have been previously incorporated into the MS diagnostic criteria.^6^ OCBs are detected through techniques such as agarose and polyacrylamide gel isoelectric focussing followed by some form of immunodetection, preferably IgG.^4^ Some research groups have investigated the value of IgM specific OCB showing some prognostic value but low diagnostic sensitivity.^26^ However, challenges exist in the reproducibility and interpretation of OCB testing due to technical issues with electrophoresis, blotting procedures, and subjective pattern analysis. To mitigate these challenges, specialised laboratories with expertise in CSF analysis are essential for ensuring accurate and reliable OCB detection in clinical settings.^27^

## Determination of an intrathecal κ-FLC synthesis

κ-FLC in the CSF originate either physiologically from blood via diffusion across the blood-CSF-barrier, or from an intrathecal production under pathological conditions, such as inflammatory disorders of the central nervous system, including MS.^15^^,^^28^

From a conceptual point of view, it is necessary to determine the locally synthesised κ-FLC fraction separate from the blood-derived fraction, as is done for other proteins, such as IgG. The amount of blood-derived κ-FLC in the CSF depends on the κ-FLC concentration in peripheral blood as well as on the blood-CSF-barrier function which is determined by the CSF/serum albumin quotient (Q_alb_).^29^^,^^30^ Accordingly, the majority of studies determined κ-FLC in the CSF and serum, and subsequently calculated the κ-FLC index or the κ-FLC intrathecal fraction (IF_κ-FLC_).^14^^,^^31^ A minority of studies determined the absolute CSF κ-FLC concentration only,^32^, ^33^, ^34^, ^35^, ^36^, ^37^, ^38^, ^39^, ^40^, ^41^ arguing that the contribution of blood-derived FLC to total CSF FLC concentration is very low in cases with intrathecal synthesis, and few studies applied the CSF/serum κ-FLC quotient (Q_κ-FLC_).^42^, ^43^, ^44^ However, in cases with low κ-FLC CSF concentrations the diagnostic performance of isolated κ-FLC measurement is significantly less compared to k-FLC index or IF_κ-FLC_,^44^^,^^45^ presumably because the relative proportion of blood-derived κ-FLC increases in the range of very low absolute CSF concentrations and can be better accounted for using the latter methods.

Due to the extensive number of studies using the κ-FLC index and the lack of a clear advantage of other non-linear functions,^43^^,^^46^, ^47^, ^48^ the κ-FLC index is the preferred measure of intrathecal κ-FLC synthesis.

## Diagnostic performance of κ-FLC index and cut-off issues

A systematic review and meta-analysis summarised the evidence on the diagnostic accuracy of the κ-FLC index in comparison to OCB, including 32 studies with approximately 3300 patients with CIS/MS and 5800 control subjects.^31^ The diagnostic sensitivity of κ-FLC index (weighted average) was 88% (range 52%–100%) with a specificity of 89% (69%–100%), while OCB had a sensitivity of 85% (37%–100%) and a specificity of 92% (74%–100%).^31^ The analysed studies included heterogeneous cohorts of patients (e.g., applying different MS diagnostic criteria, including variable amounts of patients with CIS) as well as heterogenous control populations (e.g., inflammatory and/or non-inflammatory neurological disease controls). These and other methodological differences between studies explain some of the reported variabilities in terms of diagnostic sensitivities and specificities. Despite between-study and within-study heterogeneity, the above-mentioned meta-analysis clearly showed the diagnostic accuracy of κ-FLC index and OCB is identical on a 95% confidence level at a statistical power of 99%.^31^ Furthermore, the concordance between κ-FLC index and OCB has been reported at a rate of close to 90%.^17^^,^^49^

A wide range of κ-FLC index cut-off values between 2.4 and 20 has been published, probably due to outliers and small number of patients in some studies. Considering only studies with more than 100 patients and/or controls, the range of κ-FLC index cut-off was 4.6 and 12.5, with an interquartile range of 5.9 and 7.5. A weighted mean κ-FLC index cut-off could be determined at 6.1.^31^ Methodological differences between studies might account for some variability of κ-FLC index cut-off values. E.g., including patients with other inflammatory neurological diseases as control group rather than patients with pure non-inflammatory neurological diseases might result in higher discriminatory cut-off values (or, vice versa, when a fixed cut-off is applied, in different diagnostic sensitivities and specificities).^35^ Also, cut-off values vary depending on whether the focus is to increase diagnostic sensitivity or diagnostic specificity.^50^ The impact of laboratory methods on κ-FLC measurements is discussed below. Although there are κ-FLC assays approved for its use in CSF and serum, the manufacturers do not specify κ-FLC index cut-off values. A recent multicentre study revealed that using a site-specific cut-off or a fixed cut-off (of 6.1) did not impact the classification of present or absent κ-FLC synthesis.^49^ Applying this cut-off will likely result in a robust diagnostic performance which is further substantiated by a recent study in patients with inaugural optic neuritis including a high number of NMOSD and MOGAD cases as controls.^51^ Nevertheless, as a general rule, each laboratory should verify this cut-off value.

## Prognostic value of κ-FLC

The McDonald criteria were not developed to differentiate MS from other diseases and should only be applied when the likelihood of MS is considered high.^6^ For this reason, most diagnostic studies based on subjects with clinically isolated syndromes (CIS)/early MS assessed prognosis besides diagnostic properties. The main studied outcomes were the risk of future inflammatory activity and of disability accrual. This is also the case with available evidence on κ-FLC, with most studies assessing the κ-FLC index.

### Future inflammatory activity

Overall, κ-FLC index is significantly higher in subjects who present a second attack or fulfil the diagnostic criteria for MS compared to those without further disease activity until last follow-up.^7^^,^^17^^,^^45^ When considered together with other known risk factors for MS, κ-FLC index above commonly studied cut-offs at the time of the CIS is an independent predictor of presenting a second attack or of fulfilling MRI DIT or the combination of dissemination in space (DIS) and dissemination in time (DIT).^17^^,^^45^ Importantly, κ-FLC index values are similar in CIS and radiologically isolated syndromes (RIS) and are also predictive of T2 lesion accrual as well as onset of clinical symptoms in the latter.^45^^,^^52^

A potential advantage of measuring κ-FLC over OCB is that their quantification may also be useful as a predictor of high disease activity in terms of time to relapse and higher number of relapses during follow-up even within the OCB positive population.^7^^,^^53^

The predictive value of combining κ-FLC with other biomarkers has been investigated. The combination of low and high values of κ-FLC index and serum neurofilament light (sNfL) z scores showed the highest probability of a second attack during the first year of follow-up in subjects in whom both biomarkers were high, followed in descending order by decreasing sNfL z scores and κ-FLC index either in combination or as isolated values.^54^ Looking at treatment failure (relapse, two or more new T2 lesions on an MRI performed 6 months after treatment initiation, or progression independent of relapse activity—PIRA) only isolated κ-FLC index or in combination with sNfL was predictive but not sNfL alone.^55^ An overview of studies investigating the prognostic value κ-FLC is shown in Supplemental Table S2.

### Disability accrual

Evidence regarding the value of κ-FLC to predict disability accrual is inconsistent. In terms of EDSS progression some authors found a predictive value of κ-FLC index,^53^ whereas others did not.^7^^,^^56^ More data is needed to understand the role of κ-FLC in this context.

## Neurofilament light chain

Neurofilaments as structural axonal proteins have attracted much attention as biomarkers of axonal damage in various neurological diseases. Out of several subunits, the neurofilament light chain (NfL) has received the most attention. In MS, NfL was mostly investigated for monitoring of disease activity and treatment response, showing that elevated levels are associated with a worse prognosis, higher disease activity on MRI as well as clinically, and levels decrease on treatment.^57^ NfL derives from axons of both the peripheral and central nervous systems and as such, is not specific to MS, but in the absence of another condition causing axonal damage, can reflect the degree of damage due to MS. Recent technical improvement allows reliable detection of serum/plasma levels even at very low concentrations, i.e., in the lower pg/mL range.^57^ Well known confounders are age, kidney dysfunction and body mass index (BMI). NfL concentrations increase with age and kidney disease and decrease with BMI.^58^

There are many publications on NfL in context with monitoring disease activity and drug response recently summarised in a review paper.^59^

Commercial NfL assays are most often used to determine prognosis for early MS and for disease monitoring. NfL measurement does not aid in the diagnosis of MS, but very high levels could perhaps point to a different neurological condition speaking to the first criterion of McDonald—“no better explanation”. In a retrospective analysis performed in several German MS centres including 369 patients with CIS according to the 2010 McDonald criteria, the added value of sNfL levels to gadolinium enhancing MR lesions (Gd+ lesions) and OCB was evaluated regarding the re-classification of these patients to CIS or relapsing-remitting MS (RRMS) according to the 2017 McDonald criteria.^60^ The diagnostic accuracy for RRMS versus CIS increased statistically significantly by 5% (from 79.4 to 84.3%) when sNfL levels above the 90th percentile were considered additionally to OCB positivity and Gd+ MRI scans.

There are some published sNfL and pNfL reference populations including healthy persons and or other control groups from various observational studies. The reference ranges were determined on different assay platforms, and all were adjusted for age.^61^, ^62^, ^63^, ^64^, ^65^, ^66^ In one group, NfL was also corrected for BMI.^61^ Applying these reference limits to a MS population included in the Multiple Sclerosis Partners Advancing Technology and Health Solutions network (MS PATHS), elevated NfL concentrations (i.e., diagnostic sensitivity) were found in 3.7–30.9% using the 95th percentile cutoff and in 2.5–14% using the 97.5th percentile cut-off.^67^ The diagnostic performance of a diagnostic test can be described by receiver-operating characteristics (ROC) which is a trade-off curve between diagnostic sensitivity and specificity.^68^ Ideally, the sum of both should be greater than 1.5, so even if the diagnostic specificity of a test would be 1 (i.e., 100%) the minimum sensitivity should be 0.5 (i.e., 50%) which is clearly not the case for NfL in MS. This and the limited evidence overall are why, no matter in which matrix it is measured, NfL is not useful for diagnostic purposes in suspected MS.

## CSF analyses and serum markers in paediatric onset multiple sclerosis (POMS)

The role of CSF analysis in POMS mirrors the importance in adults undergoing investigation for MS. The presence of CSF OCBs contributes to the diagnostic criteria for MS, irrespective of age at onset.^6^ The presence of CSF OCBs is one of the most powerful predictors of MS diagnosis in children presenting with an acute demyelinating event.^69^ The proportion of POMS with positive CSF OCBs varies across studies, ranging from 60 to 96%.^70^ A higher proportion of POMS with CSF OCBs has been reported in more recent studies, likely owing to testing for myelin oligodendrocyte glycoprotein (MOG)-IgG leading to removal of patients now confirmed to have MOGAD.^71^

In a study of 21 patients with POMS, increased levels of κ-FLC monomers were found in all 21 patients^70^ as well as in clinically isolated syndrome (one of whom was confirmed to have MS during follow-up) and in a patient with radiographically isolated syndrome (who had a family history of MS). Elevated κ-FLC was not entirely specific to MS and was seen in a few children with acute disseminated encephalomyelitis and in a child with CNS vasculitis. Testing for MOG-IgG was not reported. Of note, this study evaluated κ-FLC semi-quantitatively by western-blot. It underlines the value of κ-FLC also in POMS but no conclusions regarding cutoff values can be drawn.

A more recent study investigated 16 POMS cases and various control groups including other demyelinating syndromes (mostly MOGAD and NMOSD), encephalitis, and children without inflammatory neurological diseases.^72^ Comparing patients with POMS to the latter group the diagnostic sensitivity and specificity of a κ-FLC index >6.83 was 100% and 92%, respectively.^72^

As is well-recognised, elevations in sNfL are not specific to MS. In a study comparing sNfL between 142 POMS, 20 MOGAD and 201 paediatric healthy controls, elevated sNfL concentrations were detected in both POMS and MOGAD, were markedly higher in the MOGAD group, and were no longer elevated in samples obtained more than four months post an acute attack.^73^ Of note, sNfL levels do vary by age, decreasing from birth to age 10 years by approximately 7% per year, with stable values comparable to adult normative values by early adolescence (reviewed in^74^). However, sNfL concentrations correlated positively with relapses, shorter inter-attack intervals, increased lesion counts, with the presence of enhancing lesions, and portend a less favourable recovery from the acute attack.^75^

## The positive CSF: open issues and future research

**Fig. 3:**
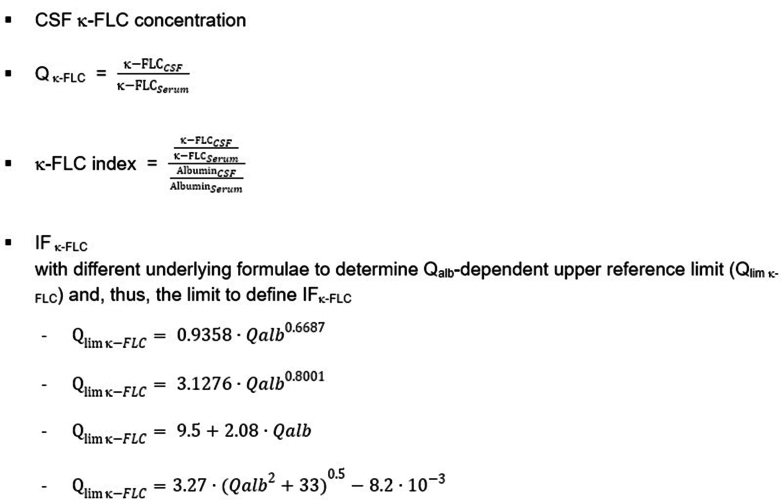
Various formulae to assess the intrathecal κ-FLC fraction. *Abbreviations*: CSF, cerebrospinal fluid; FLC, free light chain; IF, intrathecal fraction; Q_κ-FLC_, k-FLC quotient; Q_alb_, CSF/serum albumin quotient.

In summary, a positive CSF is defined by an κ-FLC index of ≥6.1 as calculated by the formula shown in Fig. 3, or by the presence of oligoclonal bands in CSF detected by isoelectric focussing followed by IgG immunoblot as shown in Fig. 2. Two or more bands in CSF that are not shown in serum (pattern type 2 and 3, Fig. 2) is the recommended cutoff and is used by most laboratories.^76^ In cases where MS is strongly suspected but the κ-FLC is not elevated, the CSF should be sent for OCB detection for confirmation and vice versa.

Studies using different platforms and assays in different sites are ongoing to confirm and establish a widely accepted cut-off to distinguish clearly negative κ-FLC synthesis from ‘grey zone’, and clearly positive κ-FLC synthesis. There will be further clarification regarding the robustness of the diagnostic κ-FLC index cutoff across laboratories using different equipment, reagents, personal qualifications, and clinical settings. Even if there is a discordance rate between OCB and κ-FLC of roughly 10% we feel that these current uncertainties are by far outweighed by the advantages of k-FLC in that it is easily performed in general labs, not requiring particular expertise, availability of (at least in the EU) certified commercial assays, automated quantification allowing several levels of cutoff for distinction between MS and other inflammatory diseases, reaching essentially 100% specificity with increasing values and last but not least much lower costs compared to OCB.

The CSF is a reservoir containing a wealth of information detailing what is happening in the CNS. In the case of an inflammatory condition like MS, it might contain elements indicative of the type of underlying inflammation, such as: involvement of complement^55^^,^^77^; indicators of the stage of MS via analysis of microRNA and targeted proteomics^78^; cytokine profiles that predict prognosis or response to therapy^79^; a specific biomarker for MS such as CD138^80^; or a marker such as osteopontin, that can be correlated with the degree of disability or cortical atrophy.^81^

NfL is an important axonal damage biomarker in both CSF and serum, but is not specific at all for diagnosing MS. It can offer some prognostic information in cases where it is unclear if a patient will have further attacks, such as in optic neuritis^82^ and has many other demonstrated values that are beyond the scope of this manuscript.^83^

An accurate diagnosis of MS is vital to the management of the condition as the treatment has become more complex with an evolving precision that depends on patients having the illness. Clinical characteristics along with imaging and fluid markers such as CSF all play important roles in defining the disease. It is unlikely in the future that it will be possible to avoid examining all aspects of disease, including the CSF, unless an exclusive diagnostic (e.g., genetic) marker is found. CSF analysis in the diagnostic work up for MS can both help in excluding non-MS mimic conditions, as well as in diagnosing MS with accuracy.

## Technical note: comparative assay methodologies using turbidimetry vs. nephelometry

κ-FLC have been studied primarily using reagents amenable to nephelometry or turbidimetry. Both methods rely on the principles of light scattering and absorption, respectively, to determine the amount of FLC present. The first commercial assay was FreeLite (Supplemental Table S1). There are different versions of the reagents for use in nephelometric or turbidimetric platforms. FreeLite has shown a drift in serum measurements over the years attributed to a kappa calibrator issue,^84^, ^85^, ^86^ binding non-specifically to alpha-1-antitrypsin and other proteins. This drift has not shown to affect measurements in CSF as medical decision points studied have remained stable.^35^^,^^38^ When using the same reagents in different platforms, results compare well in serum^87^^,^^88^ however different reference intervals have been proposed for specific instruments,^89^^,^^90^ and specific populations. Patients with significant renal impairment may benefit from a distinct new reference interval for serum FLC concentration,^91^ likely due to the presence of FLC dimers that accumulate and aggregate non-covalently in the absence of effective renal clearance. There is evidence that the κ-FLC index is not affected by renal impairment. The κ-FLC index compensates for renal function effects by factoring in serum κ-FLC concentration.^30^

κ−dimers are less likely than lambda to form dimers given their shorter constant domain and more hydrophilic C-terminal region. Lambdas have a more hydrophobic constant region making them prone to self-associate via non-covalent interactions. Dimerisation is more common in conditions like AL amyloidosis (∼70% of cases are lambdas) and light chain deposition disease, and less common in multiple myeloma. In CSF, dimerisation is unlikely, but plausible if the CSF has a low protein content and ionic strength, without a significant clearance route. Dimers are better recognised by assays that employ polyclonal antibodies (FreeLite^92^ and potentially Sebia), as monoclonal antibodies used as reagents would have narrower epitopes and could reduce dimer detection, also making those assays less likely to need separate reference intervals for renal impairment.^93^, ^94^, ^95^

Newer reagents for ELISA are now available, which may enable smaller laboratories to adopt FLC testing for clinical use without need for dedicated instrumentation and use less sample volume for testing in comparison to nephelometry and turbidimetry.

As new assays have become available, they have been analysed against the performance of FreeLite as the comparator method. There is no reference material available for FLC. In serum, analytical comparisons between different methods showed linear regression slopes ranging from 0.6 to 0.9 depending on the cohorts used for studies (e.g., monoclonal gammopathy of undetermined significance, healthy subjects, multiple myeloma).^96^, ^97^, ^98^, ^99^ Fewer comparisons have been carried out in CSF across methods.^100^ Studies in MS evaluating the κ-FLC index did not find significant sensitivity differences across nephelometry or turbidimetry subgroups compared to OCB, excluding the platform type impact on the calculation.^31^

External quality assessment programs for free light chains in CSF and serum are available from multiple proficiency testing programs. Certified κ-FLC assays should be used that are approved for CSF and serum even if these assays are only certified in some regions of the world (see Supplemental Table S1). CSF can be relatively easy validated in labs that already run serum κ-FLC tests. The above mentioned κ-FLC index cut-off of 6.1 can be applied to all CSF certified assays, after validation by local laboratories.

## Contributors

F.D. delivered the topic presentation. T.C. and X.M. drafted meeting agendas. F.D., G.A., B.L.B., T.C., X.M., and M.S.F. participated in discussion and reaching consensus. F.D., H.H., G.A., B.L.B., S.G., H.T., M.A.W., and M.S.F. were involved in preparation of the first draft of the manuscript. All authors reviewed the draft manuscript and have read and approved the final manuscript.

## Declaration of interests

Florian Deisenhammer has participated in meetings sponsored by or received honoraria for acting as an advisor/speaker for Alexion, Almirall, Biogen, BMS, Sanofi, Horizon, Janssen, Laurea Group, Medwhizz, Merck, Novartis Pharma, Neuraxpharm, Roche, Sandoz, and Teva. His institution has received research grants from Biogen, Novartis Pharma, and Sanofi. He is review editor of Frontiers Neurology.

Harald Hegen has participated in meetings sponsored by, received speaker honoraria or travel funding from Amgen, Bayer, Biogen, Bristol Myers Squibb, Janssen, Merck, Novartis, Sanofi-Genzyme, Siemens, Teva; and received honoraria for acting as consultant for Biogen, Bristol Myers Squibb, Novartis, Roche, Sanofi-Genzyme, and Teva.

Georgina Arrambide has received compensation for consulting services, speaking honoraria or participation in advisory boards from Roche, Horizon Therapeutics, and Bristol Myers Squibb; and travel support for scientific meetings from Novartis, Roche, ECTRIMS, and EAN. G.A. serves as editor for Europe of the Multiple Sclerosis Journal – Experimental, Translational and Clinical journal; and as a member of the editorial and scientific committee of Acta Neurológica Colombiana; is a member of the International Women in Multiple Sclerosis (iWiMS) network executive committee, the European Biomarkers in Multiple Sclerosis (BioMS-eu) steering committee, the MOGAD Eugene Devic European Network (MEDEN) steering group, and the Platform Adaptive Trial for remyelination and neuroprotection in mUltiple Sclerosis (PLATYPUS) steering committee.

Brenda Banwell has served as a consultant to Novartis, Sanofi, Teva Neuroscience, and Biogen in the design of clinical trials for pediatric multiple sclerosis. She has served as a central imaging reviewer for clinical trials by Novartis and Roche and received grant funding from the National Institutes of Health, National Multiple Sclerosis Society, and Multiple Sclerosis Canada.

Timothy Coetzee is CEO of the National MS Society, a sponsor of the International Advisory Committee on Clinical Trials in MS.

Sharmilee Gnanapavan has received support for conference attendances and speak fees/honoraria from CMSC, Jansen Cilag, MS Academy, Novartis, Sanofi Genzyme and Roche. She has also received Grant support from Sanofi Genzyme, Merck and Takeda.

Xavier Montalban's institution has received compensation for lecture honoraria and travel expenses, participation in scientific meetings, clinical trial steering committee membership, or clinical advisory board participation in recent years from Abbvie, Actelion, Alexion, Bial PD, Biogen, Bristol-Myers Squibb/Celgene, EMD Serono, Genzyme, Hoffmann-La Roche, Immunic Therapeutics, Janssen Pharmaceuticals, Medday, Merck, Mylan, Nervgen, Neuraxpharm, Novartis, Peervoice, Samsung-Biosys, Sandoz, Sanofi-Genzyme, Teva Pharmaceutical, TG Therapeutics, Excemed, Medscape, ECTRIMS, MSIF, and NMSS or any of their affiliates.

Hayrettin Tumani reports honoraria for acting as a consultant/speaker and/or for attending events sponsored by Alexion, Bayer, Biogen, Bristol-Myers Squibb, Celgene, Diamed, Fresenius, Fujirebio, GlaxoSmithKline, Horizon, Janssen-Cilag, Merck, Novartis, Roche, Sanofi-Genzyme, Siemens, Teva, and Viatris.

Maria Alice Vieira Willrich discloses the following relationships/activities/interests: research support from Sebia Inc., Binding Site, and Siemens for research studies; royalties from Binding Site for the intellectual property “Quantifying Monoclonal Therapeutic Antibodies by LC-MS/MS”; advisory board service for Myeloma360 in 2021; honoraria from ADLM for the 2024 annual scientific meeting; support from ADLM for attendance of annual scientific meetings and from the College of American Pathologists from 2018 to 2023; a patent issued for “Quantifying Monoclonal Therapeutic Antibodies by LC-MS/MS”; membership and vice chair position in the Diagnostic Immunology and Flow Cytometry Committee at the College of American Pathologists from 2018 to 2023; division chair for infectious diseases and immunology at ADLM; and associate editor for the Clinical Chemistry Journal.

Mark S. Freedman received research support from Sanofi-Genzyme Canada; speaking fees from Hoffman La-Roche, Novartis, EMD Inc.; and honoraria and consulting fees from Amgen, Astra Zeneca, EMD Inc./EMD Serono/Merck Serono, Find Therapeutics, Hoffman La-Roche, Novartis, Sandoz, Sanofi-Genzyme, Sentrex, Teva Canada Innovation. He also has received compensation for service on advisory boards and corporate boards from Amgen, Astra Zeneca, Autolus, Bayer Healthcare, Celestra Health, EMD Inc./Merck Serono, Find Therapeutics, Hoffman La-Roche, Neurogenesis, Novartis, Sanofi-Genzyme, Sentrex, Setpoint Medical; and compensation for service on data safety monitoring boards from Abata Therapeutics, Celltrion, Hoffman La-Roche, and Moderna.
