# Supplementary material for: Positive cerebrospinal fluid in the 2024 McDonald criteria for multiple sclerosis
Source: eBioMedicine. 2025 Sep 17;120:105905. doi: 10.1016/j.ebiom.2025.105905 (PMC12571584; doi:10.1016/j.ebiom.2025.105905)
Supplement: Supplemental Table S1 [file mmc1.docx]

**Supplemental Table 1.** Various FLC assays available around the world

| **Manufacturer (Reagent name)** | **Different platforms using the reagents** | **Characteristics of the assay** | **Comments** | **Authority Approval**  **Status** |
| --- | --- | --- | --- | --- |
| The Binding Site (FreeLite) | Nephelometry   - Siemens BNII - Siemens BN Prospec - Beckmann Immage   Turbidimetry   - Optilite - SpaPlus - Roche cobas | Sheep anti-human polyclonal antibodies | Testing may be performed by nephelometry or turbidimetry on the various platforms.  Different dilutions employed for each instrument. Different dead volume requirements for the platfoms may affect labs choice for CSF measurement. | EU: CE-certified for CSF and serum  US: FDA approved serum only |
| Siemens Healthineers (N Latex) | Siemens BNII  Siemens BN Prospec  Atellica neph630 | Cocktail of monoclonal antibodies | All instruments are nephelometers, no difference in reference intervals for serum assay in renal disease. | EU: CE-certified for CSF and serum  US: FDA approved serum only |
| Sebia Inc. (Sebia FLC) | Any ELISA reader | Rabbit polyclonal antibodies | Same reagent set is versatile to be used with many automated or manual platforms, very low sample volume required for testing. | Serum only, not approved for CSF anywhere |
| Roche (Diazyme) | Roche cobas | Polyclonal  (kappa: rabbit and goat) | Turbidimetric method, automated instrumentation suitable for high volume laboratories | Serum only, not approved for CSF anywhere |
| Seralite | Lateral flow immunoassay with a specific instrument  ADxLR5 absorbance reader | Cocktail of monoclonal antibodies | Lateral flow assay with quick turn-around-time for serum tests | Serum only, not approved for CSF anywhere |

Table modified from reference 96.
