# Supplementary material for: Positive cerebrospinal fluid in the 2024 McDonald criteria for multiple sclerosis
Source: eBioMedicine. 2025 Sep 17;120:105905. doi: 10.1016/j.ebiom.2025.105905 (PMC12571584; doi:10.1016/j.ebiom.2025.105905)
Supplement: Supplemental Table S2 [file mmc2.docx]

**Supplemental Table 2 Articles on the prognostic value of κ-FLC.**

| **Author** | **Type of cohort** | **n** | **Age** | **Follow-up** | **Type of outcome** | **Definition of outcome** | **Results** |
| --- | --- | --- | --- | --- | --- | --- | --- |
| Berek  (unicentric) (7) | CIS/early MS (McDonald 2017) | 88 | 33±10*** | 47 (38-48) months** | Future inflammatory activity | Survival analysis for  second attack  by κ-FLC index | κ-FLC index per increase of 10:  HR 1.13, p=0.009  Baseline κ-FLC index >100:  2x higher probability of CDMS within 12 months |
| Arrambide (unicentric) (16) | CIS/early MS (McDonald 2017) | 214 | 34 (28- 41)** | 5.9 (2.9–10.0) years** | Future inflammatory activity | Survival analyses for  second attack  as well as  fulfilment of DIS and DIT on MRI^  by κ-FLC index | Second attack:  κ-FLC index ≥5.9: HR 2.0 (0.9-4.3), p=0.068  κ-FLC index ≥6.6: HR 2.1 (1.1-4.2), p=0.035  κ-FLC index ≥10.61: HR 1.5 (0.8-2.6), p=0.210  DIS and DIT on MRI^:  κ-FLC index ≥5.9: HR 4.9 (2.5-9.6), p<0.001  κ-FLC index ≥6.6: HR 3.4 (1.9-6.3), p<0.001  κ-FLC index ≥10.61: HR 2.5 (1.5-4.3), p<0.001 |
| Levraut  (unicentric) (45) | CIS/early MS and RIS  (McDonald 2017) | CIS/early MS: n=146  RIS:  n=36 | CIS/ early MS: 38 (29-46)**  RIS: 46 (33-56) | 20 (13-33) months**  25 (16-35) months** | Future inflammatory activity | Survival analyses for  second attack,  new T2 lesions in CIS/ early MS  and  new T2 lesions in RIS  by κ-FLC index | CIS/ early MS:  Second attack:  κ-FLC index per increase of 10:  HR 1.04 (1.01-1.07), p=0.007  Not calculated with κ-FLC index 8.9: no events occurred in one of the subgroups  New T2 lesions:  κ-FLC index per increase of 10:  HR 1.06 (1.04-1.07), p<0.001  κ-FLC index >8.9:  HR 17.17 (7.41-39.79), p<0.001  RIS:  New T2 lesions:  κ-FLC index per increase of 10:  HR 1.08 (1.03-1.13), p=0.002  κ-FLC index >8.9:  HR 3.61 (1.46-8-90), p=0.005 |
| Salavisa  (unicentric) (50) | CIS/early MS  (McDonald 2010, 2017) | 28 | 30 ±9*** | 79.1 (31.4)*** | Future inflammatory activity | Univariate correlations of number of relapses at last follow-up and time to first relapse with κ-FLC quotient and κ-FLC index (Spearman σ)  Survival analysis for second attack by κ-FLC quotient | κ-FLC quotient:  Number of relapses on follow-up:  σ=0.41, p=0.031  Time until second attack:  σ=-0.58, p=0.001  κ-FLC index:  Number of relapses on follow-up:  σ=0.41, p=0.029  Time until second attack:  σ=-0.59, p=0.001  HR 4.08 (1.28-12.96), p=0.017 |
| Hegen  (unicentric) (51) | CIS/early MS  (McDonald 2017) | 86 | 33 ±10*** | 47 (38–48) months* | Future inflammatory activity | Survival analysis for second attack by κ-FLC index  Probability of freedom of relapse at 12 months combining κ-FLC index high vs lower (high: >100) and sNfL z score high vs lower (high: >3) categories | κ-FLC index (as an independent variable in addition to NfL z score):  HR 1.021 (1.010-1.032), p<0.001  κ-FLC index high + sNfL z score high: 2%  κ-FLC index high + sNfL z score low: 30%  κ-FLC index low + sNfL z score high: 70%  κ-FLC index low + sNfL z score low: 90% |
| Tortosa-Carreres  (bicentric)  (52) | MS  (McDonald 2017) | 137 | 35 (17)* | 4 (3.3) years* | Future inflammatory activity | Survival analyses for therapeutic failure^#^ by κ-FLC index >130 ^§^ | Multivariate model including age and  κ-FLC index >130:  HR 2.69 (1.35-5.37), p=0.005  Multivariate model including EDSS and κ-FLC index >130:  HR 2.67 (1.32-5.40), p=0.006  Model combining κ-FLC index >130 and high sNfL (>Q3) as one variable:  HR 3.11 (1.5–6.5), p =0.002 |
| Berek (unicentric)  (7) | CIS/early MS (McDonald 2017) | 88 | 33 ±10*** | 47 (38-48) months** | Disability accrual | EDSS ≥3.0 | No estimations: no differences in κ-FLC values between subjects with EDSS ≥3.0 vs <3.0 |
| Salavisa  (unicentric) (50) | CIS/early MS  (McDonald 2010, 2017) | 28 | 30 ±9*** | 79.1 (31.4)*** | Disability accrual | Univariate correlations of time until confirmed disability progression¶ and MSSS at follow-up with κ-FLC quotient and κ-FLC index  (Spearman σ)  Survival analysis for confirmed disability progression by κ-FLC quotient | κ-FLC quotient:  Time to confirmed disability progression: -σ=0.43, p=0.024  MSSS at follow-up:  σ=0.39, p=0.041  κ-FLC index:  Time to confirmed disability progression: -σ=0.55, p=0.002  MSSS at follow-up:  σ=0.52, p=0.004  HR 3.70 (1.44-9.50), p=0.006 |
| Arroyo-Pereiro (53) | MS (McDonald 2017) | bMS:º n=20  aMSºº: n=15 | 32 ±10*** | 19.8 (15.9-24.6)** | Disability accrual | Median (IQR) difference in κ-FLC index» or κ-FLC quotient in bMS vs aMS | κ-FLC index:  bMS: 67.27 (30.23-159.41)  aMS: 74 (37.61-332.45)  p=0.590  κ-FLC quotient:  bMS: 0.26 (0.15-0.52)  aMS: 0.39 (0.18-0.67)  p=0.640 |

According to each article:

*Median (interquartile range).

**Median (percentiles 25-75).

***Mean (standard deviation).

#Treatment failure: considered in case of relapses, emergence of ⩾2 new lesions on T2-weighted MRI (using the MRI study conducted six months after treatment initiation as a reference), or in cases of progression independent of relapse activity (PIRA).

§Two multivariate models were constructed due to collinearity between EDSS and age: one with EDSS and one with age.

±Due to the correlation observed among sNfL, age, and EDSS, these variables were excluded from the models that incorporating sNfL individually or in combination with other body fluid biomarkers.

¶ EDSS progression: increase in EDSS score of ⩾1.5 points from a baseline EDSS score of 0, ⩾1.0 point from a baseline EDSS score of 1.0–5.5 or ⩾0.5 point from a baseline EDSS score ⩾6.0, confirmed after 6 months.

ºbMS: patients with EDSS ≤3.0 at 10 years of follow-up.

ººaMS: patients with EDSS ≥6.0 at 15 years of follow-up.

»κ-FLC index from samples not necessarily taken at the time of the index event.

^Fulfilment of DIS and DIT on follow-up MRI scan (according to the 2017 revised McDonald criteria)

Abbreviations: CIS: clinically isolated syndrome; MS: multiple sclerosis; κ-FLC: kappa free light chain; HR: hazard ratio; CDMS: clinically definite multiple sclerosis; aHR: adjusted hazard ratios; 95% CI: 95% confidence intervals; MRI: magnetic resonance imaging; DIS: dissemination in space; DIT: dissemination in time; RIS: radiologically isolated syndrome; sNfL: serum neurofilament light chain; EDSS: Expanded Disability Status Scale; MSSS: Multiple Sclerosis Severity Score; bMS: benign MS; aMS: aggressive MS.

Of note: some authors used the term κ-FLC ratio in the original publications when referring to the ratio between CSF to blood κ-FLC concentrations without correction for the albumin quotient. Here we uniformly use the term κ-FLC-quotient.
